# Hair and plasma cortisol throughout the first 3 years of development in infant rhesus macaques, *Macaca mulatta*

**DOI:** 10.1002/dev.22437

**Published:** 2023-12

**Authors:** Alexander J. Pritchard, John P. Capitanio, Laura Del Rosso, Brenda McCowan, Jessica J. Vandeleest

**Affiliations:** 1 California National Primate Research Center, University of California, Davis, Davis, California, USA; 2 Department of Population Health and Reproduction, School of Veterinary Medicine, University of California, Davis, Davis, California, United States

**Keywords:** cortisol, development, HPA axis, infants, juveniles, primate

## Abstract

Cortisol expression has been demonstrated to have variation across development in rhesus macaques (*Macaca mulatta*). There exists contradictory evidence for the nature of this change, and age at which it occurs, across biological sample types. Consequently, we lack a cohesive understanding for cortisol concentrations across the development of a major human health translational model. We examined hair cortisol concentrations over the first 3 years of life for 49 mother-reared infant macaques from mixed-sex outdoor units at the California National Primate Research Center. For 48 of these subjects at infancy, 1 year, and 2 years, we obtained plasma cortisol samples for response to a stressor, adjustment to prolonged stress, and response to dexamethasone injection. Hair cortisol concentrations decreased dramatically between 3 and 10 months, followed by relative stability up to the final sampling event at around 34 months of age. Plasma cortisol showed within-year consistency, and consistency between infancy and year 1. We document variability in the infant plasma cortisol samples, especially in percent change between samples 1 and 2. Our plasma cortisol results indicate that infants possess the physiological capacity to effectively inhibit the release of cortisol when stimulated, as effectively as later responses in juveniles. Age-related changes in hair cortisol parallel findings indicating a large decline in the weeks following postparturation.

## 1 ∣ INTRODUCTION

The expression of high levels of cortisol during infancy has been posited to alter and impede neuronal development (Wingfield & Romero, 2015). During development, however, cortisol remains essential for numerous bodily functions, including metabolic activity and acute, as well as chronic, stress responses (Sapolsky et al., 2000). The necessity of functional cortisol production at these critical ages introduces a complex trade-off whereby short-term benefits of a responsive hypothalamic–pituitary–adrenal (HPA) axis can have life-altering consequences if cortisol production is high enough. Indeed, the deleterious consequences of high cortisol production have been posited as the probable ultimate mechanism for the stress hyporesponsivity period observed in altricial organisms, such as rats and mice (Sapolsky & Meaney, 1986; Wingfield & Romero, 2015).

How does this trade-off function in a long-lived cognitively complex organism, such as a primate? Many primates do not exhibit the stress hyporesponsivity period, but have been posited to be behaviorally buffered against high cortisol production via maternal support (Sanchez et al., 2015). We focus on rhesus macaques (*Macaca mulatta*) as a frequently used translational health model. Several studies have examined the normal development of cortisol production in infant macaques, though many rely on plasma cortisol. Here, we briefly review these findings, before synthesizing them into expected growth trajectories.

Studies on cortisol development in rhesus macaques have primarily relied on measuring basal or stress-induced plasma cortisol. Basal plasma cortisol has been described as stable between 14 and 30 days of age, based on 16 control infants (Champoux et al., 1989). In a small group of mother-reared infant rhesus macaques sampled monthly throughout the first 24 weeks of life, however, Clarke (1993) reported a gradual decrease of plasma cortisol concentration in response to an injection of adrenocorticotropic hormone. Similarly, Capitanio et al. (2005) reported significantly higher stress-induced plasma cortisol in younger infants (89–100 days old) relative to older infants (101–130 days old) in a sample of over 750 subjects, but reported no changes in stress-induced plasma cortisol between small groups of ten 3- and ten 6-month-olds. McCormack et al. (2009) reported lower basal plasma cortisol in ten 1-month-old infants relative to the same infants at 6 months in a healthy control group of rhesus macaques, though this difference was not formally tested. Similarly, later work showed a gradual increase in basal plasma cortisol across the first 6 months of life in 42 cross-fostered infant rhesus macaques (McCormack et al., 2022). After 6 months, Koch et al. (2014) reported a steady increase in circulating basal cortisol for rhesus macaques, as well as cortisol levels in response to a stressor, until after 2 years of age. After this time, however, between 24 and 30 months of age, Koch et al. (2014) reported a marked decrease in basal plasma cortisol. Basal cortisol levels showed intraindividual consistency across years, especially in temporally adjacent sample periods, while stress-associated levels had reduced stability.

Taken together, these findings indicate the following pattern: a reduction of basal (McCormack et al., 2009) and stress-induced (Capitanio et al., 2005; Clarke, 1993) plasma cortisol in the first 6 months of life, though this dynamic is not uniformly expressed (as evident in McCormack et al., 2022). Within this period, there may be periods of stability in very young infants or these gradual changes might not be discernable in reduced timeframes (e.g., 2 weeks, as in Champoux etal., 1989). Rhesus macaques are expected to exhibit adult-like patterns of diurnal cortisol secretion starting at 5 months, but still show immaturity until 12 months (Raper et al., 2013; Sanchez et al., 2015). Even so, basal plasma cortisol has been posited to increase from 6 months up until 2 years of age, after which it has been shown to decrease before stabilizing (Koch et al., 2014).

Many of the aforementioned studies relied on plasma cortisol, which often quantifies circulating bound and unbound cortisol, but is sensitive to recent experiences, diurnal rhythms, energetic expenditure, energy intake, and acute stressors. Other biological samplings, such as hair cortisol or fecal glucocorticoid metabolites, accrue longer periods of circulating baseline levels of cortisol that can diminish the influence of short periods of heightened cortisol (e.g., in response to acute stressors). Hair cortisol has been associated with unbound cortisol levels that match weekly (Davenport et al., 2006; Kapoor et al., 2014) or multi-day (Colding-Jørgensen et al., 2020) patterns of physiological accrual rather than rapid measures that show changes in responses within minutes, such as plasma cortisol (Sapolsky et al., 2000).

A handful of relevant publications provide insight into the developmental trajectory of nonhuman primate hair cortisol. In rhesus macaques, changes in hair cortisol throughout development have been explored less than changes in plasma cortisol. McCormack et al. (2022) simultaneously measured plasma and hair cortisol across the first 6 months of life; hair cortisol showed a marked decline in two point samples 6 months apart despite a gradual increase in plasma cortisol in the same timeframe. Importantly, initial hair cortisol samples were acquired 2 days after birth and were over four times as high relative to samples from the same subjects 6 months later. Similar findings were reported in samples taken from infant rhesus macaques within the first 2–4 days of life, with infant hair cortisol concentrations almost an order of magnitude higher than that of their own mothers—albeit with greater individual variation (Kapoor et al., 2014); maternal and infant cortisol were not correlated. Grant et al. (2017), similarly, reported heightened hair cortisol at birth in 22 indoor nursery-reared infant pigtail macaques (*Macaca nemestrina*). These concentrations surpassed those of their own mothers, sampled at the time of delivery; infant hair cortisol then exhibited a general decline over the first 6 months of life, stabilizing during the remaining sample events up until 10 months. These development trajectories, however, are not immutable to environmental changes, as shown by Dettmer et al. (2014), who also demonstrated that infants and juveniles had higher hair cortisol relative to adults. One-year-old juveniles, however, had comparable hair cortisol to infants in high-density housing (170.81 ± 10.76 and 172.94 ± 12.36, respectively), despite differences between these two age groups in low-density conditions (70.88 ± 3.14 and 140.92 ± 13.53, respectively). As Grant et al. (2017) emphasized, housing or rearing conditions might importantly alter the slope of these trajectories and account for observed differences in hair cortisol during development despite a highly canalized process.

Research on HPA activity during development in humans is more resolved. Hair cortisol samples taken at birth parallel findings in macaques, with heightened concentrations at birth for infants born at term (Stoye et al., 2021). These heightened concentrations were not present in preterm infants, an observation that is linked to the term infants’ in utero exposure to heightened fetal cortisol 2–3 weeks prior to term delivery (Fowden et al., 1998; Stoye et al., 2021). Patterns of circulating “basal” cortisol, likely, remain unchanged up to 5–8 months of age, before decreasing—though there is some evidence that this change occurs earlier (as reviewed in Jansen et al., 2010). Cortisol reactivity decreases over time, with individuals older than 6 months having an attenuated response relative to responses at a younger age. Importantly, this is not true for all individuals as some individuals may show extreme responses to psychological stressors that do not elicit a response among individuals in the general population (as reviewed in Jansen et al., 2010). After early infancy, individuals aged 12–24 months exhibit greater within-individual variability, along with heightened baseline values, relative to children aged 30–36 months (Watamura et al., 2004).

We explore how the expression of cortisol in hair and plasma changes throughout the first 3 years of development. While research has been done on the development of the HPA axis, this work is important to continue for several reasons. First and foremost, infancy is an important time of development, during which perturbations can have lifetime consequences. A baseline understanding of normative development in a large mixed-sex social group is important to have in order to understand the consequences of aberrant events or traumatic experiences. Second, we contribute data from a large group of subjects from two different birth year cohorts: 49 individuals (27 females, 22 males—though we note that sex differences typically do not emerge during these sample periods [Raper et al., 2013]). This sampling is large compared to similar studies that have values from normative control groups (11–22 individuals) (Koch et al., 2014; McCormack et al., 2009, 2022; Raper et al., 2013) that, in some circumstances, have undergone craniotomies (Raper et al., 2013), cross-fostering (McCormack et al., 2022), or nursery-rearing (Grant et al., 2017). Finally, a subset of these studies often rely exclusively on plasma cortisol, which can be altered by short-term perturbations (Koch et al., 2014; McCormack et al., 2009; Raper et al., 2013). Here, we include hair cortisol as a measure of circulating cortisol concentrations over a longer period of time, but also include measures of cortisol reactivity, sustained response, and suppression. We posit two general hypotheses:

### 1.1 ∣ Accrued cortisol levels will change throughout development, with greater changes within the first 6 months of life

While developmental trajectories have been previously documented, there is not a consensus as to the direction of this change. Therefore, we have contrasting predictions: (a) infants less than 6 months of age will have higher hair cortisol, relative to later periods of development (Capitanio et al., 2005; Clarke, 1993; Gesquiere et al., 2005; Grant et al., 2017; Jansen et al., 2010; McCormack et al., 2022); (b) infants less than 6 months of age will have lower hair cortisol, relative to later periods of development (McCormack et al., 2009, 2022; Sanchez et al., 2015); or (c) there will be no change in hair cortisol throughout development, the null (Capitanio et al., 2005; Champoux et al., 1989). Importantly, our work is conducted in large groups, whereby infant subjects delivered at term have access to mothers that may provide behavioral mitigation of the stress response (Sanchez et al., 2015). As this aspect of our study focuses on hair cortisol, we acknowledge that preparturation in utero exposure to high concentrations of glucocorticoids has been linked to increased cortisol samples in infants, especially immediately after birth (Fowden et al., 1998; Grant et al., 2017; Kapoor et al., 2014; Stoye et al., 2021). Growing evidence, however, suggests that hair cortisol is unlikely to be retained in the hair on the order of months (Colding-Jørgensen et al., 2020; Kalliokoski et al., 2019; Kapoor et al., 2018). Furthermore, maternal effects have been posited to have an uncertain but putative influence on hair cortisol (Grant et al., 2017; Hinde et al., 2015), which could be discernable via shifts during transitions during the process of weaning. Finally, prepubertal changes could be posited to be associated with biobehavioral adjustments (Kulik et al., 2015a, 2015b), relevant as individuals near 3 years of age in the tail-end of our study.

### 1.2 ∣ Cortisol reactivity, in response to a challenge, will change throughout development

Younger infants will have greater variability in plasma cortisol when responding to a stressor, relative to older infants (Jansen et al., 2010). To our knowledge, this former phenomenon has not been formally asserted and tested in nonhuman primates despite data that indicate a similar developmental pattern may be present in nonhuman primates (McCormack et al., 2022), including evidence of high variability in infant hair cortisol (Grant et al., 2017; Kapoor et al., 2014). There is no indication, however, that older individuals will exhibit greater reactivity. Other published data, however, do not show variation across developmental windows, lending support that there might not be changes in cortisol reactivity (Koch et al., 2014; McCormack et al., 2009; Raper et al., 2013). This discrepancy with human data (reviewed above) is important to resolve for comparability of rhesus macaque infants as a translational model for human development.

## 2 ∣ METHODS

We had two cohorts (2016 and 2017) represented by 49 subjects with hair cortisol samples (cohort 1: 13 females, 11 males; cohort 2: 14 females, 11 males); for 48 of these subjects, we also obtained plasma cortisol samples, less one male in cohort two. Subjects were mother-reared animals housed in the same half-acre outdoor housing unit within a large mixed-sex social group of 120–156 (annual mean = 133.5; with a mean sex ratio of 2.05 F:M) monkeys across the four study years (2016–2019). Outdoor housing units contained A-frame structures, benches, and pole perches available for ad libitum animal use. Chow biscuits were provisioned into feeders affixed cage-side, twice daily in the early morning and mid-afternoon providing liberal availability of chow during the day. Subjects were also provisioned with fresh produce weekly. Animals were checked every morning for injuries or maladies and removed from the cage to administer necessary veterinary care.

Two data collection events were used to acquire biological samples: BioBehavioral Assessments (BBA) and round-up events. Round-ups are routine health checks and biological sampling events for the entire group provided every 6 months within the social housing unit. Animal subjects are corralled into a large temporary holding area, then directed toward a chute where they are separated individually using a trapping comb. Animal subjects, over 6 months of age, were anesthetized with an age–weight appropriate dose of injectable ketamine. Veterinary staff screen animals for injuries and draw the necessary biological samples, while trained research staff acquire any necessary hair samples. For BBA, our animal subjects were removed from the outdoor housing unit and placed into holding cages (60 × 65 × 79 cm; Lab Products, Inc.) within auditory, but not visual, range of several peers. Subjects were held for 25 h and administered several behavioral tests in the holding cages and a testing cage (38.7 × 52.1 × 47.0 cm) in an adjacent testing room (Capitanio, 2017; Capitanio et al., 2006). Sample collection is detailed below. Briefly, during BBA events a trained technician collected four plasma cortisol samples from infant subjects, and three samples from the same subjects at 1 and 2 years of age. For infants, we also acquired hair samples from our subjects during this time. All protocols for this study were approved by the Institutional Animal Care and Use Committee of the University of California, Davis, CA.

### 2.1 ∣ Hair cortisol

#### 2.1.1 ∣ Sampling

Initial hair samples were taken during routine BBA (Capitanio, 2017; Capitanio et al., 2006) when the 49 infants were 3–4 months of age (102 ± 10*SD* days). Hair was sampled after the second plasma sample was collected at the end of the first testing day, to avoid interfering with the results of other BBA tests. Subsequent samples were taken during round-up events starting at January of the year following infant birth and continuing at approximately 6-month intervals thereafter until subjects were almost 3 years of age, resulting in five round-up sampling events per subject (average days of age at each round-up: 290 ± 27*SD*; 461 ± 24*SD*; 651 ± 24*SD*; 826 ± 26*SD*; 1022 ± 24*SD*). In summary, the first sample collection event occurred asynchronously for subjects at their individually scheduled BBA events, while the remaining events occurred synchronously during a total of seven routine round-up events (five for each subject across the two cohorts). If aggregating by collection events, relative to each subject’s first event (i.e., BBA, round-ups 1–5), we obtained a mean of 47.67 hair samples ± 1.21*SD* per sampling event for a total of 286 hair cortisol samples.

#### 2.1.2 ∣ Assays

We ground hair samples, used methanol for extraction, and ran enzyme immunoassays (Salimetrics) as previously validated and reported elsewhere (Vandeleest et al., 2019). Samples that exceeded an intrasample coefficient of variation (CV) of 15% were rerun; we discarded one sample prior to analysis that was erroneously not rerun, despite an invalid reading. Of the retained samples (*N* = 285), the average intraassay sample CV was 2.86%. We ran two quality controls per plate, each in duplicate. Within the quality controls, the average intrasample CV was 4.08%, though two quality control samples from distinct plates had high CVs (59.53% and 13.01%) and much higher than expected cortisol values in one or both wells; however, CVs for each plate’s second quality control sample were acceptable (1.61% and 2.70%, respectively). The interassay CV was 8.17% after excluding the two quality control samples with high CVs.

#### 2.1.3 ∣ Analyses

To assess the developmental trend of cortisol expression in hair, we fit a Bayesian regression model (brm) using Stan (Bürkner, 2017) with age, temperature data as a control, subject sex, subject birth cohort, and random effects for subject identifiers. Temperature data were obtained from the University of California Davis’ Russell Ranch weather station (UCD/NOAA Climate Station: USC00042294 [Atmospheric Science Program, Land Air & Water Resources, University of California Davis, 2023; Menne et al., 2012]), which is less than half a mile from the outdoor animal housing units. We simplified the raw temperature data into daily maximums and minimums. Fecal glucocorticoid concentrations have been shown to covary with environmental factors, such as temperature, due to differences in thermoregulation or energy expenditure in wild baboons (*Papio* spp.) (Gesquiere et al., 2011; Weingrill et al., 2004). Similar outcomes have been reported in a small sample of outdoor-housed Japanese macaques (*Macaca fuscata*) (Takeshita et al., 2014). As hair samples reflect cortisol concentration over weeks, we fit several windows of aggregate temperature data to the hair cortisol values to select the temporal aggregate window that best fit the data. Because temperature was not a central focal point, we found this approach the most appropriate for finding a best-fit window to select the appropriate timeframe of mean-maximum and mean-minimum temperature for each sample. An average of maximum and minimum temperature for the 7 days prior to each hair sample provided the best fit, relative to temporal windows of 14, 21, or 28 days.

We fit a brm using Stan through *brm()* (Bürkner, 2017) using R (v4.2.2) (R Core Team, 2022) in the R-Studio IDE (v2023.06.1+524) (RStudio Team, 2018) for statistical analyses. We constructed models using a lognormal distribution. We included age as a smoothed thin-plate spline, with additional fixed effects for scaled maximum temperature over the prior week, subject sex, and subject cohort as controls, as well as subject identifier as a random effect. Minimum and maximum temperature values were highly correlated (*r*_294_ = .91) and, to avoid collinearity, we only selected maximum temperature as its inclusion showed improved fit over full models with minimum temperature. We scaled temperature and age by two standard deviations (Gelman, 2008). Two outliers were excluded to improve model fit. These outliers were both from the third round-up sampling period and were highly distant from the mean for cortisol relative to surrounding data points.

We ran the model with weakly informative (default) priors; we used 4 chains and 6000 iterations with a warm-up of 1000 and a thin of 2, resulting in 10,000 post-warmup draws. We assessed alternative biologically meaningful models using expected log pointwise predictive densities *loo_compare()* to determine whether formulas improved model fit. We graphically examined model fit using the outputs of both *loo()* and *pp_check()*, and visual inspection of fit was acceptable; we also examined *pairs()* plots for evidence of collinearity (Figure S1). Alternative model formulas or smooth classes did not improve fit. We also inspected predicted data using *epred_draws()* and *fitted()* to assess model fit. Including interactions between sex and age did not improve fit. The final model had no run divergences, but 282 of the total count had good Pareto *k* values, with the single remaining sample having *k* < 0.7. Thus, we retained the model specification. Rhat values were also assessed for model fit and were all equal to 1. Effective sample size estimates were between 3879 and 6640 for bulk_ESS and between 4930 and 7139 for tail_ESS. We report model summary statistics, in addition to probability of direction values (PD) for fixed effects, which provide interpretive similarity to *p*-values for researchers more familiar with frequentist analyses (Henzi et al., 2021). We used the bayestestR package where two-sided *p*-values approximate PD values as follows: 2 × (1 – PD/100) (Makowski et al., 2019); though PD values are not true cutoffs (McElreath, 2018). We emphasize, however, that smooth parameter estimates are indicative of spline variance (“wiggliness”) and, therefore, meaningful differences along the spine were assessed based on a lack of overlap in credible intervals. Finally, we quantified individual repeatability in hair cortisol estimates following Hertel et al. (2020). We divided the resulting model’s subject identifier random effect variance by total variance to obtain an estimate of across individual repeatability (Hertel et al., 2020).

### 2.2 ∣ Plasma cortisol reactivity

#### 2.2.1 ∣ Sampling

Routine BBA includes the collection of four plasma samples as standard protocol. Animals are captured in and separated from their social groups between 8:00 a.m. and 9:00 a.m.; subjects arrived at the indoor housing unit shortly after separation. The four plasma samples reflect (1) an initial response to morning relocation (sample collected at 11:00 a.m.—approximately 2–3 h after capture), (2) sustained response to separation and relocation (4:00 p.m.), (3) infant response the next morning after the administration of an intramuscular injection of dexamethasone (500 μg/kg), a synthetic glucocorticoid that impairs endogenous cortisol output (dexamethasone administered immediately following the second sample; third blood sample obtained at 8:30 a.m.), and (4) infant response to the intramuscular administration of adrenocorticotropic hormone (ACTH) (2.5 IU/kg), the main stimulant for cortisol’s release from the adrenal (ACTH administered immediately following sample 3; sample 4 was obtained 30 min following the injection of ACTH). We replicated the BBA at 1 and 2 years of age. During these replicate events, we took the first three plasma cortisol samples, omitting the ACTH-stimulated sample. Later BBA events, at year 2, had fewer overall tests relative to infant BBA tests, but the overall timing of test schedules and total BBA duration were uniform. Infant and 1-year-old subjects were hand caught and manually restrained by an indoor team during blood draws; however, the year 2 juveniles were restrained in the holding cages by a different crew for arm draws. We obtained a mean of 46.89 ± 0.60*SD* samples per type (samples 1, 2, and 3) and sampling event (infancy, year 1, and year 2) for a total of 422 samples.

#### 2.2.2 ∣ Assays

Plasma samples were assayed using previously validated quantitative competitive immunoassays (Siemens Healthcare Diagnostics) (Vandeleest et al., 2019). These samples were processed identically to the standards held for the over 5000 subjects that BBA has been conducted on (Capitanio, 2021). Samples that failed to meet these standards were re-assayed.

#### 2.2.3 ∣ Analyses

Rather than test mean absolute cortisol values between the groups, we additionally quantified percent changes between samples within each year. Thus, we quantified response to the sustained testing of the BBA as a percentage of the second sample relative to the first sample. We also quantified chemically induced declines as a percentage of the third sample relative to the second sample. We term these sustained response and percent suppression throughout, respectively. We did not explicitly test for sex and cohort differences, primarily due to prior research emphasizing small effect sizes of such differences in BBA plasma sampling (Capitanio et al., 2005).

## 3 ∣ RESULTS

### 3.1 ∣ Developmental trends in hair cortisol

**FIGURE 1:**
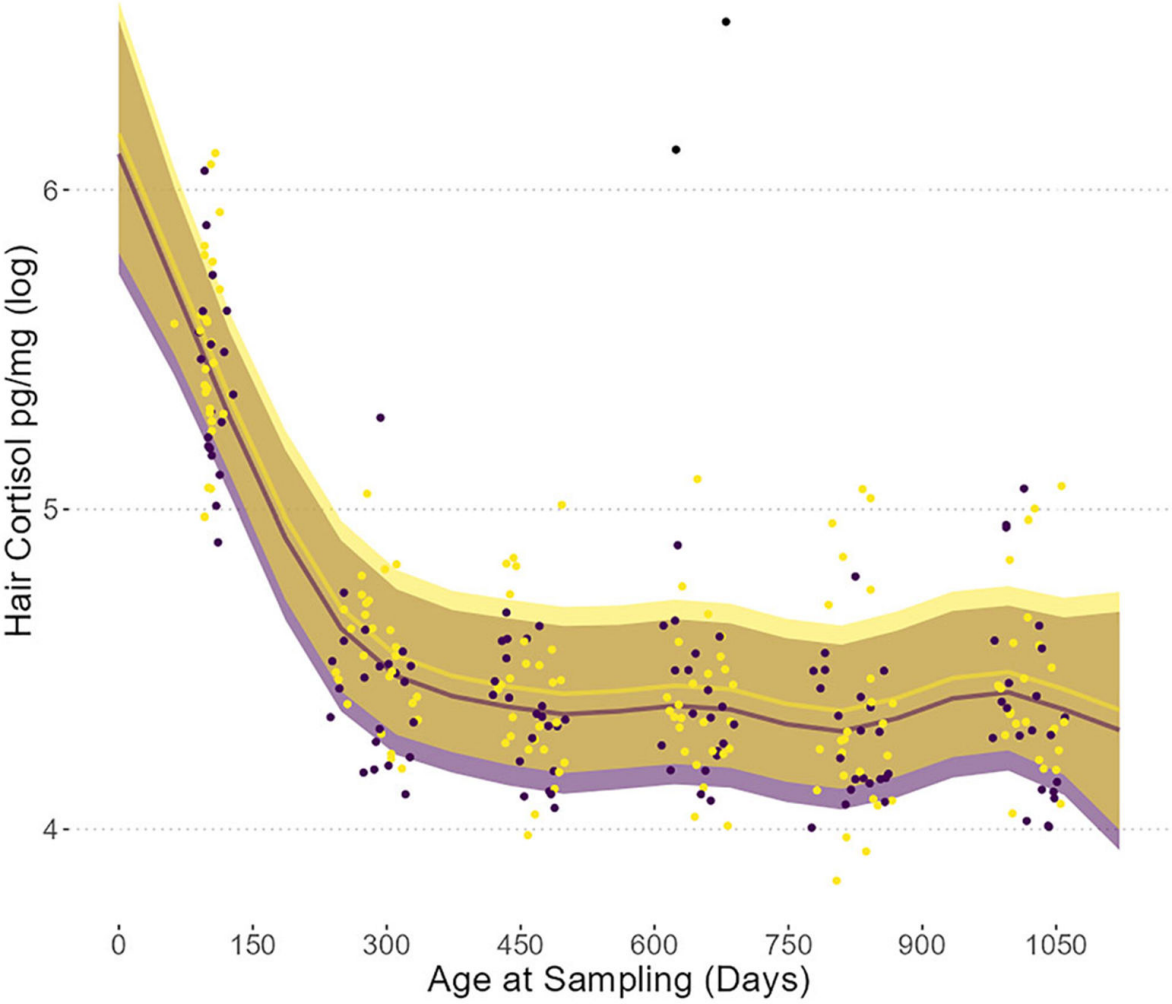
Shifts in hair cortisol values over time. Age (days) of individual at sampling is on the *x*-axis, and hair cortisol (pg/mg) is on the *y*-axis (note the log scale). Points represent individual samples males (purple) and females (gold), with the two omitted outliers (black—upper center). The smooth includes standard errors (95% credible intervals) plotted from full model predictions of cortisol values from the brm using epred_draws with a mean maximum temperature.

Hair cortisol concentrations showed a rapid initial decline followed by stability in samples after the second age sampling event (i.e., from 290 ± 27*SD* to 1022 ± 24*SD* days). The final model was a better fit relative to a model with only the random effect (elpd difference = −181.80 ± 16.5). We observed a large early decrease in hair cortisol (pg/mg) between the first sampling event (102 ± 10*SD* days) and the second (290 ± 27*SD* days) (Table 1; Figure 1), consistent between both cohorts and sexes. Due to this sharp decrease, the relationship between cortisol and age was nonlinear with the later sampling points having uniformly lower cortisol relative to the first sampling event. Thus, we fit a brm with age as a smoothed spline, with fixed effects for maximum temperature over the prior week, cohort, and subject sex as controls, as well as subject identifier as a random effect (Table 2; Figure 1). The smoothing fit for age supported a nonlinear relationship in its association with hair cortisol (age smooth estimate = 2.12, 95% lower credible interval [CI] = 1.06, upper CI = 3.92), which distinguished the youngest individuals as having especially high cortisol (Figures 1, S2, and S3), relative to all the later sampling events. Hair cortisol remained stable across the remaining sampling events.

Minimum and maximum temperature values were highly correlated (*r*_294_ = .91) and, to avoid collinearity, we only selected maximum temperature for inclusion as a more powerful predictor for these data. The addition of a smooth for temperature did not improve fit relative to its inclusion as a linear predictor. The linear predictor for temperature was not associated with hair cortisol (estimate = 0.03, 95% lower CI = −0.12, upper CI = 0.17; PD = 64.45%). Males had similar hair cortisol relative to the female reference group (estimate = −0.06, 95% lower CI = −0.15, upper CI = 0.17; PD = 92.01%), and cohort 2 had similar hair cortisol relative to the cohort 1 reference group (estimate = 0.03, 95% lower CI = −0.05, upper CI = 0.12; PD = 75.70%).

### 3.2 ∣ Hair cortisol repeatability

Hair cortisol showed moderate individual repeatability over the ages sampled. Following Hertel et al. (2020), we calculated among individual repeatability from the random effects by extracting the standard deviations for random effect terms from the posterior, then squaring these values to obtain variance before dividing each random effect by the total random variance in the model. We found that 21.71% of the variance (with 95% intervals of 9.28%–34.41%) was attributable to subject identifier, as compared to 78.29% (65.59%–90.72%) for residual variance. Because these values are linked to the *y*-intercept and our data were centered, we reran the model with the data centered to the mean age of individuals at the latest and earliest sampling points. The resulting repeatability estimates were consistent with those reported here (all within ± 1.00%). Thus, there is moderate variance attributable to among individual repeatability in our hair cortisol samples.

### 3.3 ∣ Plasma cortisol reactivity

**FIGURE 2:**
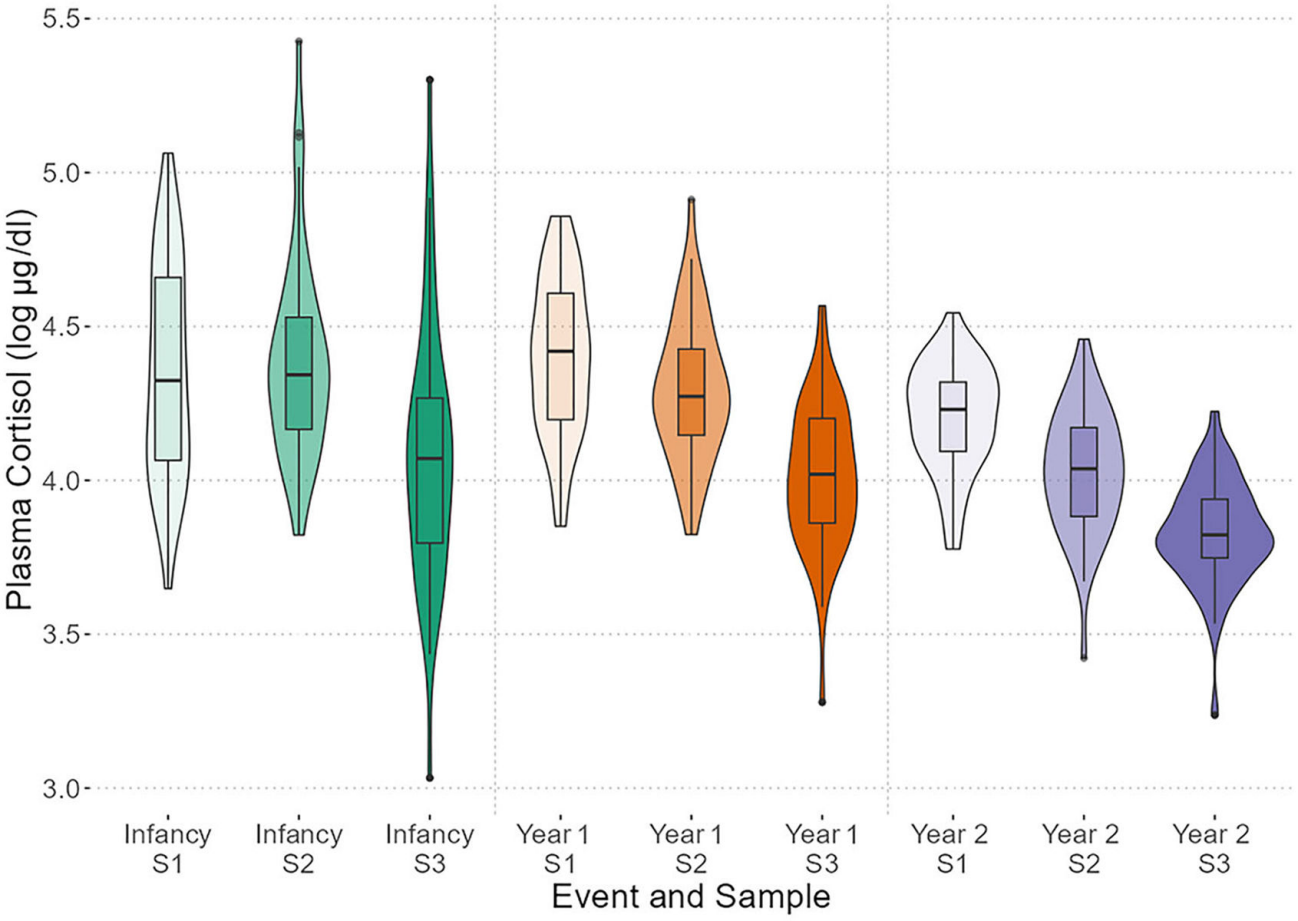
Violin plot, with nested boxes, for plasma cortisol values (μg/dL, logged) on the *y*-axis across sampling events and types. Color is used to separate sampling event, while hue emphasizes the sampling type (S1 = initial response; S2 = afternoon CORT; S3 = morning after dexamethasone).

We observed within-year consistency between plasma cortisol values and moderate consistency across infant and year 1 samples despite high variability within the infant samples. Plasma cortisol samplings showed high-to-moderate cross-correlations within their respective years (Figure S4). Within the year 1 and 2 events, we observed moderate correlations between their first and second samples, but the third samples were moderate to poorly correlated. Across years, we observed moderate correlations between the infant event samples and the respective year 1 event samples, but poor correlations between the infant event samples and the respective year 2 event samples. The infant event samples appeared to have higher variability in their raw values across the three sample types, relative to later events (Table 3; Figure 2). To determine whether this variability was significant, we ran a Brown–Forsythe test with pairwise comparisons, adjusted for multiple comparisons using a Holm–Bonferroni correction. The overall test was significant (B–F statistic = 18.71, *df*_num_ = 8, *df*_denom_ = 237.93, *p* < .001), indicating that samples and years had significantly different variances. Pairwise comparisons between the relative samples (e.g., 1 vs. 1, 2 vs. 2) indicated that all three infant samples were not different in variance from the respective year 1 samples (*p*-values > .05), but all three year 2 samples differed from their respective infant and year 1 samples (*p*-values < .05) (Table S1). Thus, year 2 samples had uniformly reduced variability relative to the earlier time points.

**FIGURE 3:**
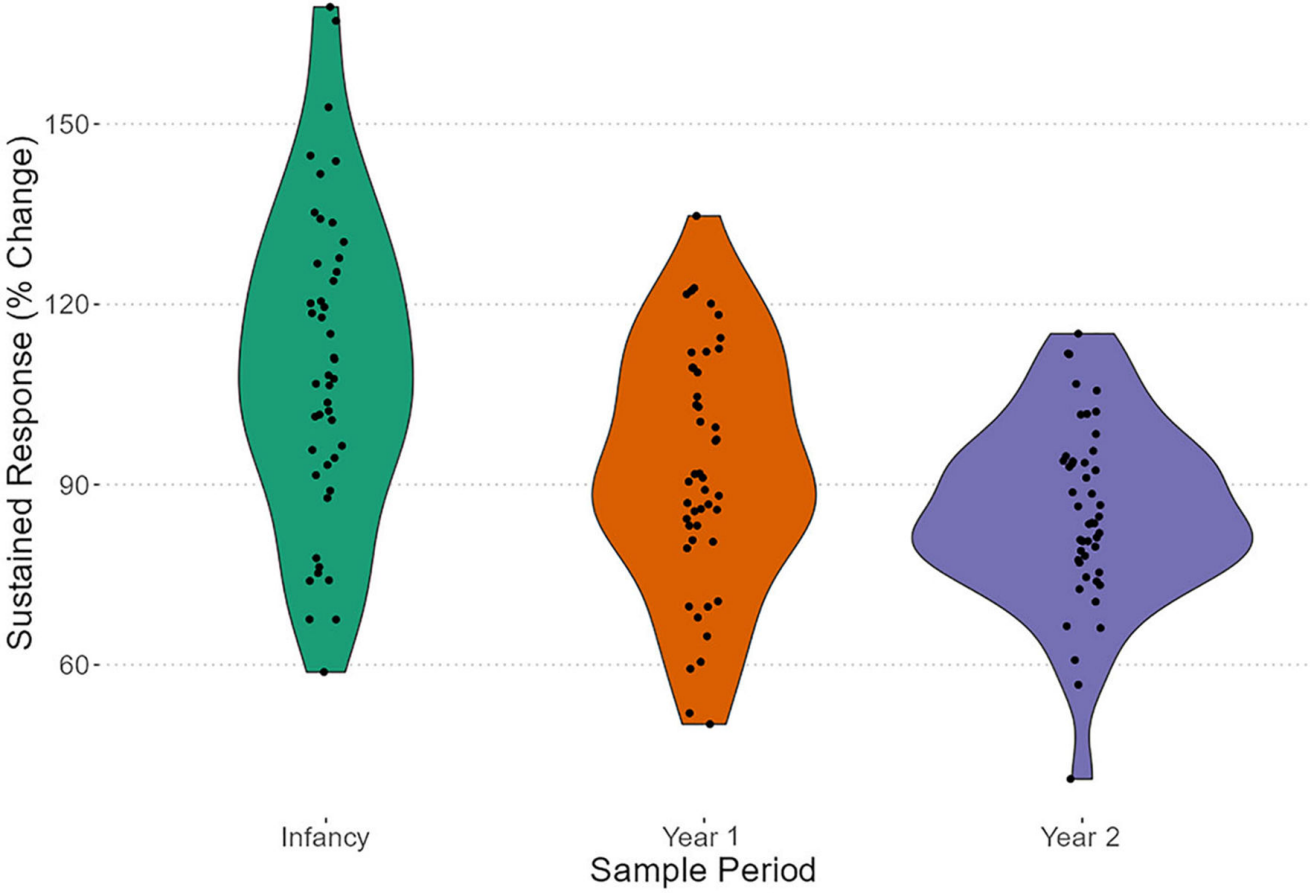
Sustained response—percent change to sample point 2 from sample point 1. Violin plots with jittered points; color to emphasize year of sampling.

**FIGURE 4:**
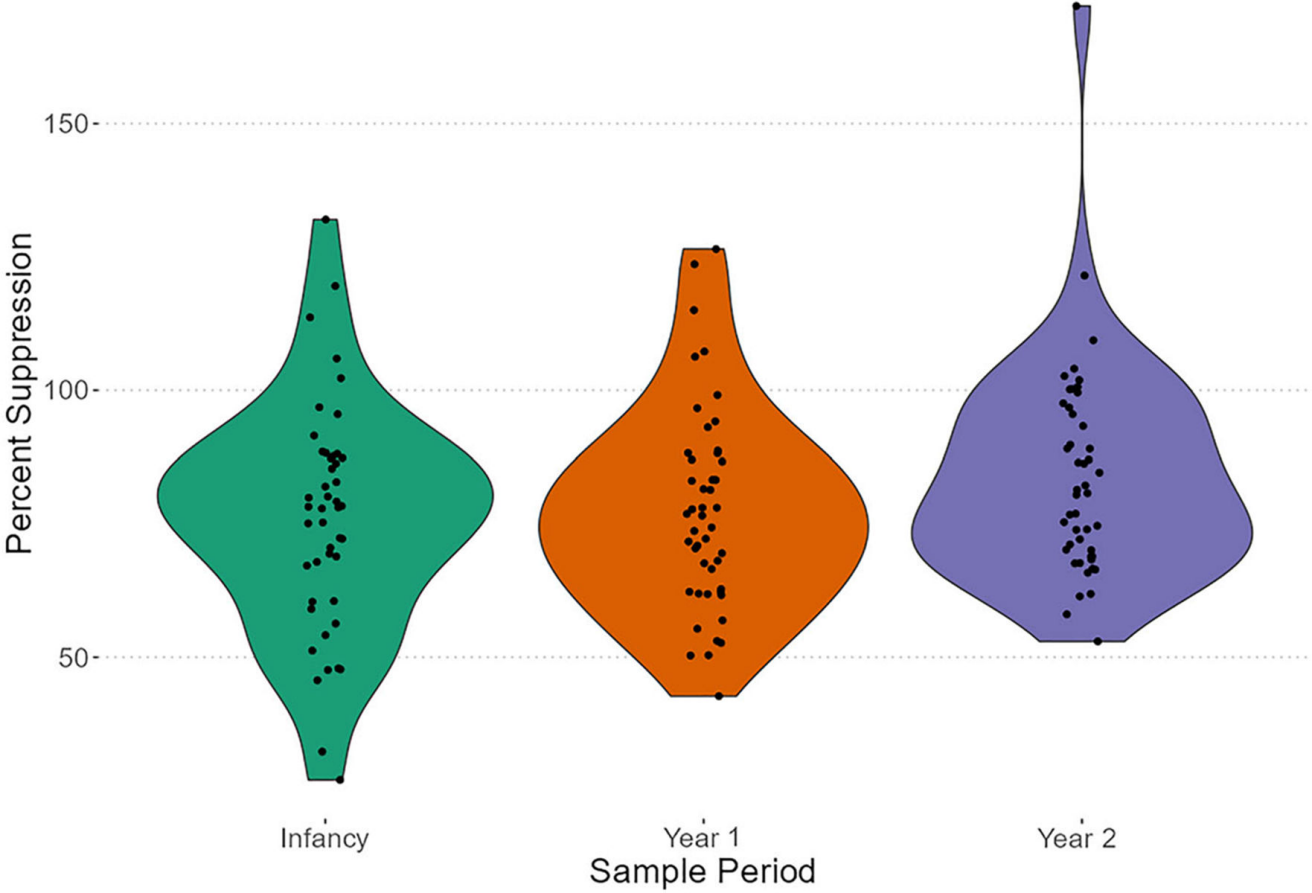
Percent suppression of endogenous cortisol output by dexamethasone—percent change to sample point 3 from sample point 2. Violin plots with jittered points; color to emphasize year of sampling.

To determine the effectiveness of subjects’ responses, we examined percent sustained response between samples 1 and 2, and percent suppression between samples 2 and 3. A decrease in the sustained response was more pronounced for older individuals, as evident by reduced variation across older individuals relative to when they were younger (Figure 3). To determine whether this variability was significant, we ran a Brown–Forsythe test. The overall test was significant (B–F statistic = 16.84, *df*_num_ = 2, *df*_denom_ = 113.78, *p* < .001), indicating that sustained response exhibited significantly different variances across years. We then examined whether there were mean differences between the groups via a one-way analysis of variance (ANOVA) using Welch’s adjustment to compensate for unequal variances. The ANOVA indicated significance (*F* = 15.36, *df*_num_ = 2, *df*_denom_ = 85.73, *p* < .001); pairwise comparisons using *t*-tests with nonpooled *SD* with a Holm–Bonferroni correction indicate that sustained response means differed significantly between infants’ and year 1 juveniles’ samples (*p* = .001) as well as infants’ and year 2 juveniles’ samples (*p* < .001), but not between year 1 and year 2 juveniles’ samples (*p* = .06). Overall differences for percent suppression were not present (Figure 4). We confirmed this assertion with a Brown–Forsythe test that was not significant (B–F statistic = 1.98, *df*_num_ = 2, *df*_denom_ = 135.31, *p* = .14). Similarly, there were no differences between group means via a one-way ANOVA (*F* = 1.99, *df*_num_ = 2, *df*_denom_ = 137, *p* = .14). We did not explicitly test differences in sex and cohort, but include visualizations that explicitly present these data by such groupings (Figures S5 and S6).

## 4 ∣ DISCUSSION

We observed altered hair cortisol levels within the first 9.5 months of life, signified by a pronounced decline from the first sampling event (102 ± 10*SD* days) to the second (290 ± 27*SD* days). These findings support a subset of a wider group of similar studies showing age-associated decreases in infant cortisol (Capitanio et al., 2005; Clarke, 1993; Gesquiere et al., 2005; Jansen et al., 2010; McCormack et al., 2022), rather than stable or increased cortisol (Capitanio et al., 2005; Champoux et al., 1989; McCormack et al., 2009, 2022; Sanchez et al., 2015). Importantly, these findings agree with directional changes observed in several studies that used measures of long-term accrual of cortisol, or glucocorticoid metabolites (Dettmer et al., 2014; Gesquiere et al., 2005; Grant et al., 2017; McCormack et al., 2022). Individuals showed relatively robust repeatability in their expression, despite these age-related declines. Younger infants, however, also had increased cortisol reactivity, which showed an age-related decline. Furthermore, individuals showed a more uniform response to a sustained challenge as they aged. We further explore these age-related changes, and present alternative explanations for why there are discrepancies between the hair and plasma cortisol results here, as well as in the wider literature.

### 4.1 ∣ Developmental trends in hair cortisol

Measures of hair cortisol show concordance with patterns of cortisol reduction throughout development in humans and nonhumans, showing a significant twofold decrease between 3 months of age and the subsequent sample at 9.5 months. This timeline is consistent with several studies involving humans and monkeys (Clarke, 1993; Gesquiere et al., 2005; Grant et al., 2017; Jansen et al., 2010; McCormack et al., 2022). For instance, McCormack et al. (2022) reported a decline between samples collected at 2 days and 6 months. Both McCormack et al. (2022) and the present study only conducted two collection events between the highest and lowest points of hair cortisol. Grant et al. (2017), however, demonstrated that a large proportion of this decline has already occurred between birth and 20 days of age. This aging period follows a preparturation period of high fetal cortisol production (Fowden et al., 1998; Grant et al., 2017; Stoye et al., 2021), but does not correlate with maternal hair cortisol (Grant et al., 2017; Kapoor et al., 2014). We acknowledge evidence that hair cortisol is not as longitudinal as previously expected (Colding-Jørgensen et al., 2020; Kalliokoski et al., 2019; Kapoor et al., 2018); however, these studies were not conducted with the extreme values pertinent to this high fetal exposure. Indeed, the hair cortisol values in the time postbirth are an order of magnitude higher than their own mothers (Grant et al., 2017; Kapoor et al., 2014). Thus, a lingering diffusion may be possible given radiolabeled hair cortisol has been found in hair up to 28 days after administration (Kapoor et al., 2018).

In conjunction to this putative diffusion, remodeling of the adrenal cortex has been posited to explain the previously observed hair cortisol decrease between 20 days and 2 months (Grant et al., 2017; McNulty et al., 1981). The adrenals remain reduced to one third of their original mass for 6 months before increasing after 200 days of age (McNulty et al., 1981). During this time, infant rhesus macaques are still nursing, but begin incorporating solid foods at approximately 3 months (Austin et al., 2013). Importantly, however, maternal cortisol is associated with infant cortisol prior to weaning (Jonas et al., 2018). Thus, one additional potential contribution to infant cortisol is maternal transmission of active cortisol via milk, which is shed in the infant’s hair. Indeed, in a study of 778 infants sampled across three birth years, a subset of 105 nursery-reared animals had significantly reduced plasma cortisol values relative to the majority of infants that had maternal access (Capitanio et al., 2005). Importantly, concentrations and shifts in maternal milk cortisol have been demonstrated to be associated with infant growth and measures of temperament (Hinde et al., 2015). It is unclear how maternal cortisol would also impact neuronal development; furthermore, the ratio of infant and maternal contributions to hair cortisol has not been published (Grant et al., 2017). Our plasma cortisol results, however, indicate that infants produce comparable or even higher cortisol concentrations to, likely weaned, 1- and 2-year-olds in response to a sustained (24 h) stressor. Thus, the majority of infant hair cortisol might be expected to primarily be produced by the infants themselves (Grant et al., 2017).

We cannot discount that the BBA sampling event itself influenced our hair cortisol results, in light of growing evidence that diffusion of cortisol into hair could occur in the span of hours (Cattet et al., 2014; Colding-Jørgensen et al., 2020; Kalliokoski et al., 2019). We deem this unlikely to be the major determining factor given hair sampling occurred on the first day of BBA testing. Furthermore, heightened hair cortisol in infant macaques has been recorded across housing and rearing conditions (Dettmer et al., 2014; Grant et al., 2017; Kapoor et al., 2014; McCormack et al., 2022).

Contrary to Koch et al. (2014), we did not find an increase in hair cortisol after the observed initial decline; Koch et al. (2014) documented increased “basal” and stress-induced plasma cortisol between samples taken at 18 and 24 months. This time period coincides with significant sociobehavioral shifts that might alter how individuals express cortisol (Kulik et al., 2015a, 2015b). Individuals at 2 years of age peak in the amount they groom, but also begin to differentiate who they groom (Kulik et al., 2015b). Furthermore, individuals had peaks in initiating aggression at 2–3 years of age (Kulik et al., 2015a), signifying a potential for behaviorally or metabolically heightened cortisol during this period. From the current data, however, it is unclear if and when hair cortisol levels change after this age, as behavioral trends continue to shift (Kulik et al., 2015a, 2015b).

We also explored individual repeatability of hair cortisol. This analysis is important to determine whether heightened cortisol might be attributed to differentially experienced stressors or uniform age-related trajectories. That is, repeatability provides more compelling evidence that this is an age-related process, rather than a dynamic occurrence. We found evidence of moderate to low among-individual repeatability in hair cortisol values. The variance estimates attributable to individual repeatability in hair cortisol for the present study (.22) were slightly lower than estimates from meta-analyses assessing the repeatability of long-term measures of cortisol: .32 (fecal, feather, or saliva samples; Schoenemann & Bonier, 2018) or .44 (fecal and hair samples; Taff et al., 2018).

### 4.2 ∣ Plasma cortisol reactivity

To assess patterns of cortisol reactivity, we assessed plasma cortisol under BBA (i.e., stressed) conditions at three ages: 3–4 months, 1 year, and 2 years. This approach is in contrast to long-term resampling of direct measures of baseline (“basal”) plasma cortisol, where studies observed slight increases in cortisol over time (McCormack et al., 2009, 2022; Sanchez et al., 2015). Similar to Koch et al. (2014), we observed individual consistency in the BBA samplings across years, as evident from our cross-correlation results. The patterns of expression, however, differed such that infant and yearling samples exhibited greater within-year variability in all three samples, relative to the year 2 samples.

Analyses of natural changes in sustained response also indicate that the younger infants had a less uniform sustained response, whereas older juveniles were more centralized in their response. This dynamic is evident in that 3- to 4-month-old infants had significantly more variable and significantly higher average changes in sustained response, relative to the same subjects at year 1 and year 2. These results parallel findings from a larger set of subjects that partitioned BBA by age at sampling (Capitanio et al., 2005). Capitanio et al. (2005) reported that their oldest sampled individuals (111–130 days) had a smaller increase in cortisol from the first to the second plasma sample, relative to younger individuals (89–110 days). There were no discernable differences, however, in our outcomes for dexamethasone-induced suppression results at the three ages.

Together, our plasma cortisol results indicate that younger individuals have the physiological capacity to regulate cortisol levels, as demonstrated by suppression, but that they do not yet have a unified response across individuals to sustained challenge. Thus, either they are continually responding to stressors, resulting in heightened variability in cortisol production, or they are unable to behaviorally accommodate stressors through coping strategies, as opposed to having a true physiological inability to effectively suppress cortisol production.

## 5 ∣ CONCLUSION

The combined plasma and hair cortisol results introduce several future directions, which cannot be investigated with the current data.

First, given that hair cortisol provides a more accumulated measure of cortisol relative to plasma, it is possible that the heightened values in rhesus macaque infants are due to sustained reactivity to daily perturbations. At this age, infants may be less adept or limited in using sociobehavioral coping mechanisms to mitigate high levels of cortisol production, as potentially evident in the lack of a uniform sustained response. Consequently, the heightened variability in the response of infants to a challenge might occur at frequent enough intervals to alter cortisol accrual in hair. These hypotheses must be reconciled, however, with the knowledge that heightened hair cortisol values have also been documented at ages prior to the samples we have reported here (Grant et al., 2017; Kapoor et al., 2014; McCormack et al., 2022). When might energetic or psychosocial perturbations regularly alter cortisol concentrations? We also must acknowledge that diurnal patterns are still not fully developed at these earlier ages (Raper et al., 2013; Sanchez et al., 2015), which may account for differences in cortisol production over longer periods than may be represented by plasma samples. Finally, we emphasize that, based on our plasma cortisol results, subjects were capable of physiologically responding to a stressor at 3–4 months of age.

Our reported values with mother-reared infants in large social groups were similar to those reported in nursery-reared pigtail macaques (Grant et al., 2017); this similarity weakens the assertion that maternal buffering is a major contributor to hair cortisol values (Sanchez et al., 2015). Similarly, maternal cortisol cannot solely be driving these dynamics. Though hair cortisol in milk-fed infants could still be naturally heightened due to cortisol dosing via maternal milk; this transfer would show an age-related decline associated with the process of weaning; prior research indicates this assertion is plausible (Capitanio et al., 2005; Hinde et al., 2015). After the initial 3- to 4-month sample, our next sample at 1 year would be around the time that weaning would be completed (Austin et al., 2013), thus we cannot explicitly test this assertion by examining whether hair cortisol shifted independent of aging during the weaning process.

We extend prior work via our plasma cortisol results that indicate that infant subjects have the physiological capacity to effectively inhibit the release of cortisol when stimulated (i.e., the animals show suppression), just as effectively as their later responses at 1 or 2 years of age. Their plasma cortisol production in response to a stressor, however, is more variable at 3–4 months and is less uniform in a sustained response to a challenge, relative to their responses at 1 or 2 years of age. This heightened variability in plasma cortisol parallels observations of high variation in hair cortisol, as evident here and previously (Grant et al., 2017; but see McCormack et al., 2022). These findings extend upon prior work by tracking hair and plasma cortisol development in the same mother-reared subjects housed outdoors in mixed-sex social units; even with these distinctions, our results closely parallel prior work.

## Supplementary Material

Supinfo

## Figures and Tables

**Table 1.** Summary of mean ± *sd* for age and raw hair cortisol concentrations across each of the hair sampling events. Note that sampling events are numbered relative to the subjects’ birth and, thus, there were more total events to sufficiently sample the two cohorts. *For the third round-up event, we have reported the raw mean, and the mean following exclusion of the two extreme outlying samples.

Sampling Event	Age (M days ±sd)	Hair Cortisol Concentrations (M pg/mg ±sd)
BBA 1	102 ±10	247 ±78
Round-Up 1	290 ±27	93 ±24
Round-Up 2	461 ±24	84 ±20
Round-Up 3	651 ±24	103 ±103 (83 ±20)*
Round-Up 4	826 ±26	79 ±26
Round-Up 5	1022 ±24	87 ±28

**Table 2.** Bayesian regression model results predicting hair cortisol values.

**Family:** Lognormal							
**Link function:** mu = identity; sigma = identity							
**Draws:** 4 chains, each with 6000 iterations; warmup = 2000; thin = 2; post-warmup draws = 8000							
**Formula:**							
Hair_Cortisol ~ Sex + Cohort + Temp_Max + s(Age) + (1∣Subject_ID)							
**Smooth Terms:**							
	Estimate	Est.Error	l-95% CI	u-95% CI	Rhat	Bulk_ESS	Tail_ESS
sds(sAge_1)	2.12	0.74	1.06	3.92	1	3879	4930
**Group-Level Effects:**							
	Estimate	Est.Error	l-95% CI	u-95% CI	Rhat	Bulk_ESS	Tail_ESS
Subject_ID sd(Intercept)	0.12	0.02	0.08	0.17	1	4126	5653
**Population-Level Effects:**							
	Estimate	Est.Error	l-95% CI	u-95% CI	Rhat	Bulk_ESS	Tail_ESS
Intercept	4.59	0.04	4.52	4.66	1	4865	5605
Sex_Male	−0.06	0.04	−0.15	0.02	1	5229	6097
Temp_Max	0.03	0.07	−0.12	0.17	1	6146	6813
Cohort_2	0.03	0.04	−0.05	0.12	1	5199	6304
**Family Specific Parameters:**							
	Estimate	Est.Error	l-95% CI	u-95% CI	Rhat	Bulk_ESS	Tail_ESS
Sigma	0.23	0.01	0.21	0.25	1	6640	7139
**Bayesian R-squared:**							
	Estimate	Est.Error	Q2.5	Q97.5			
R^2^ Conditional	0.748	0.024	0.697	0.790			
R^2^ Marginal	0.703	0.025	0.649	0.746			

**Table 3.** Summary of mean ±*sd* for age and raw plasma cortisol concentrations across each of the plasma sampling events and types. Sample types are: 1) the initial AM sample, 2) the afternoon sample after sustained individual housing, and 3) the sample after dexamethasone administration. Sampling events are numbered relative to the subjects’ birth and, thus, there were more total events to sufficiently sample the two cohorts.

Sampling Event	Age (M days ±sd)	Plasma Cortisol Concentrations (M ug/dl ±sd)		
		Sample 1: Initial	Sample 2: Sustained	Sample 3: Post-Dex.
BBA 1	103 ±10	81.89 ±28.68	86.29 ±34.38	63.68 ±31.72
BBA 2	362 ±10	84.09 ±20.91	75.30 ±18.15	56.70 ±13.2
BBA 3	725 ±11	67.88 ±11.49	57.23 ±11.24	46.61 ±7.73

## Data Availability

The data that support the findings of this study are openly available in DataDryad at https://doi.org/10.5061/dryad.rfj6q57gq.
