# Supplementary material for: Hair and plasma cortisol throughout the first 3 years of development in infant rhesus macaques, Macaca mulatta
Source: Dev Psychobiol. Author manuscript; Available in PMC 2024 Dec 1. (PMC10752380; doi:10.1002/dev.22437)
Supplement: Supinfo [file NIHMS1941411-supplement-Supinfo.docx]

**Supplementary Materials**

**Hair and plasma cortisol throughout the first three years of development in infant rhesus macaques, *Macaca mulatta***

Alexander J Pritchard^a^, John P. Capitanio^a^, Laura Del Rosso^a^, Brenda McCowan^a^, Jessica Vandeleest^a^

^a^California National Primate Research Center, University of California, Davis, California, USA


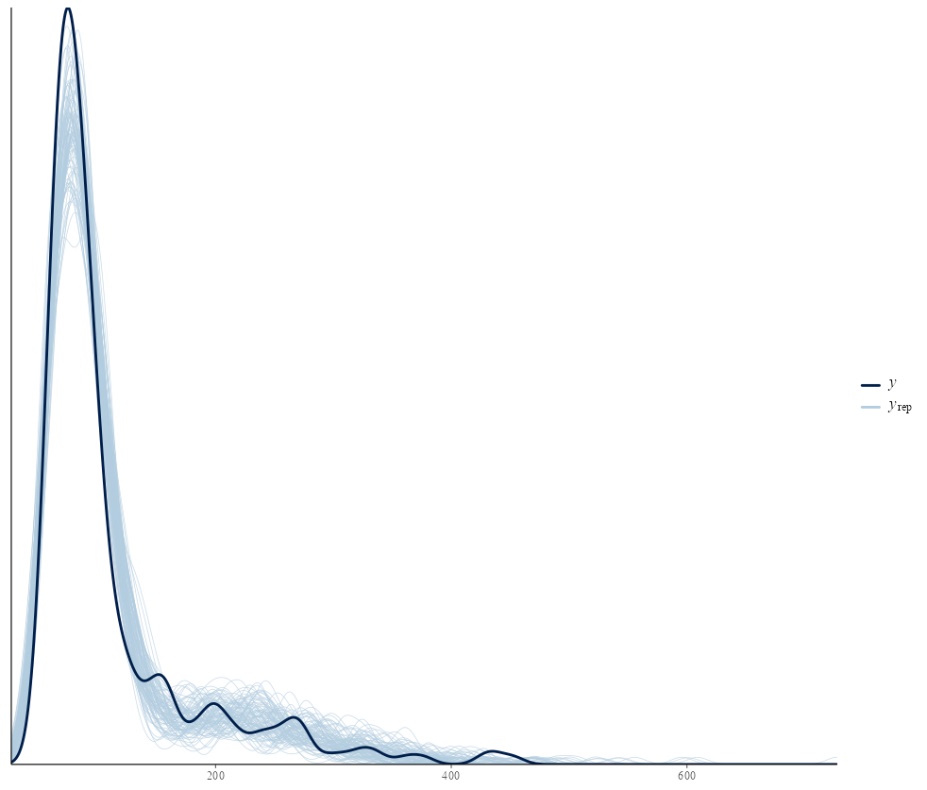


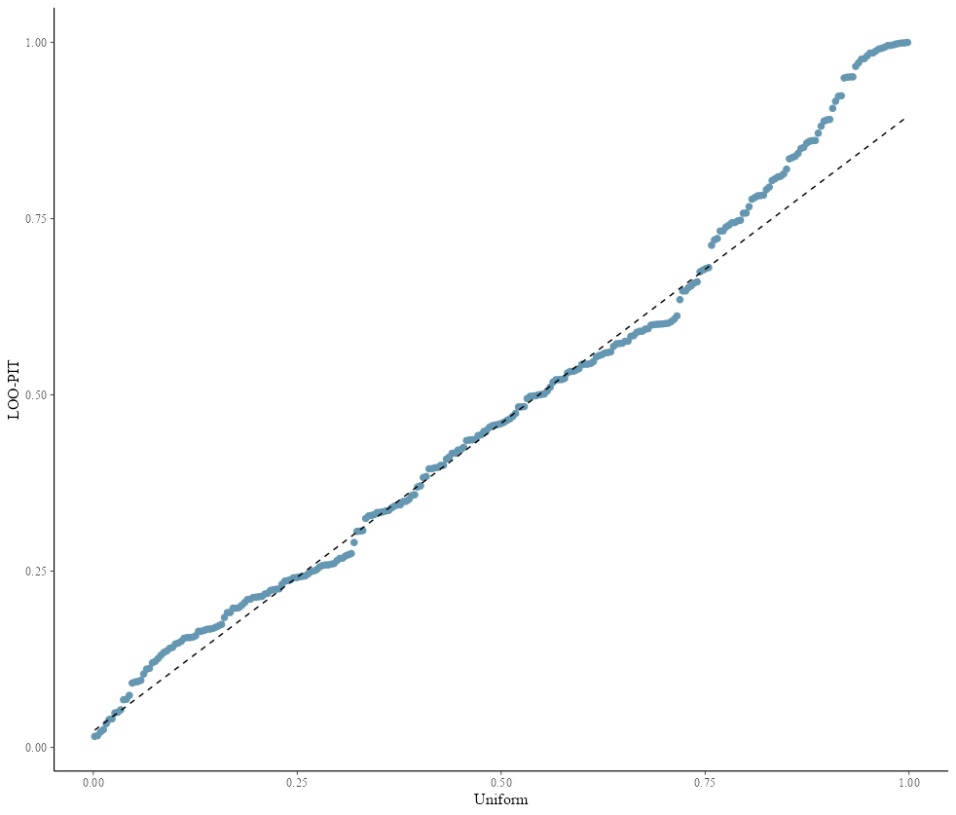


**Supplementary Figure 1**. Posterior predictive checks based on the brm. *Top:* density overlay with 100 draws; *Bottom:* leave one out probability integral transformation (loo pit) plots.

**
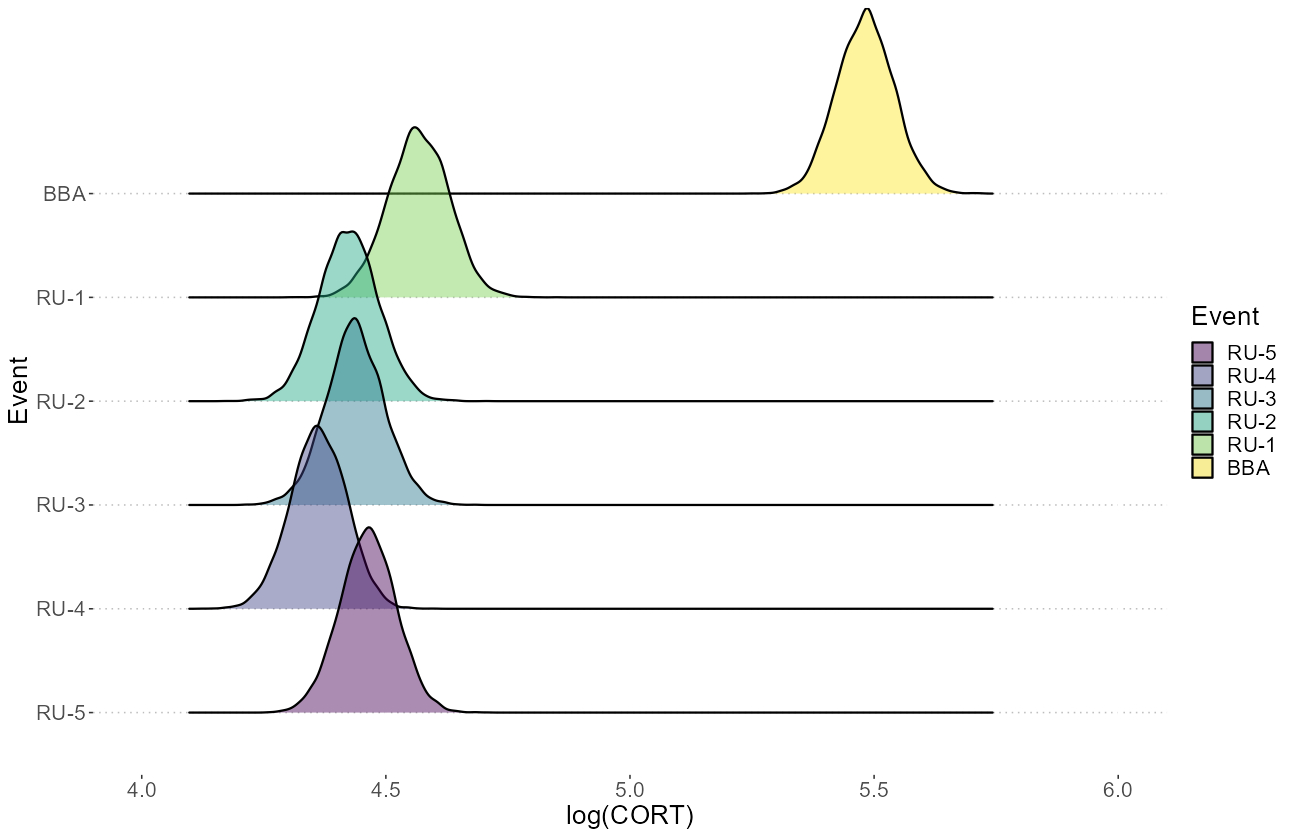
**

**Supplementary Figure 2.** Density plot of hair cortisol on a log scale (x-axis) with density samplings from the mean age of subjects at each sampling event, numbered relative to subjects’ birth (y-axis and fill color). Density plots are the estimated probabilities generated from the full fit of the model posteriors at the mean age of subjects at the point of the sampling event. The spread of the curve indicates the uncertainty of the predicted value.

**
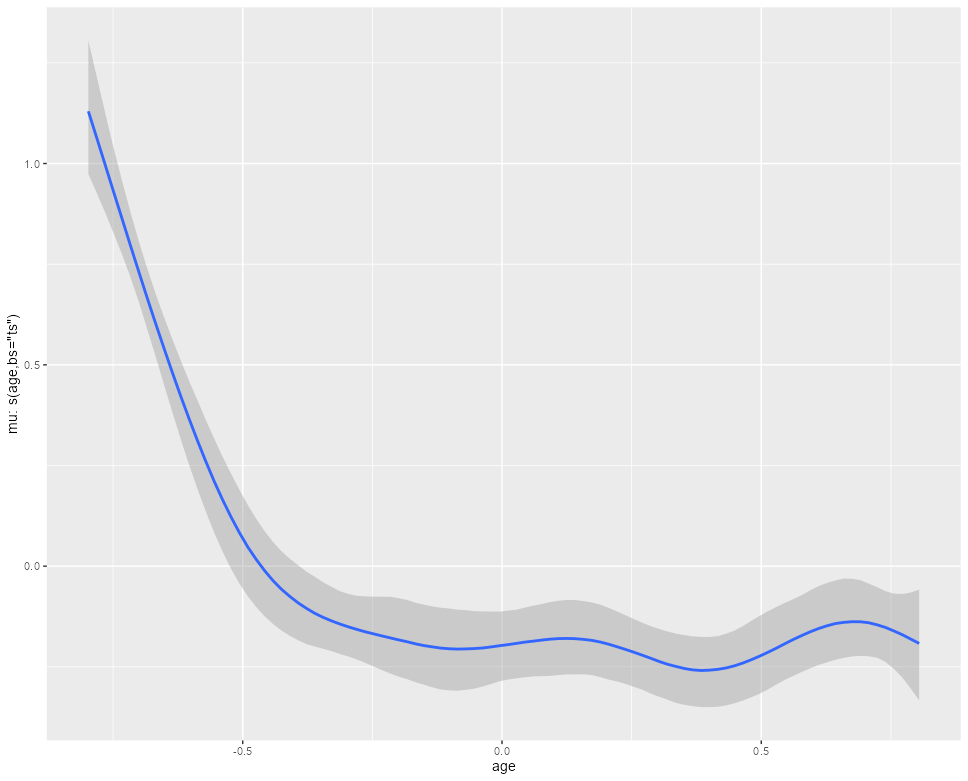
**

**Supplementary Figure 3.** Conditional smooth plot for age smooth from the final model, without additional effects.


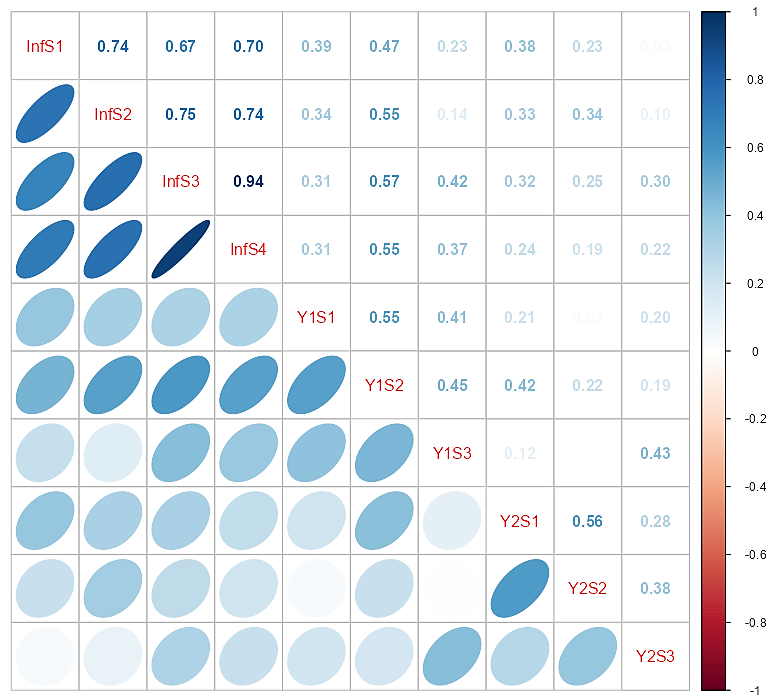


**Supplementary Figure 4**. Matrix of the correlations between each of the sample types (Initial sample = S1, Afternoon = S2, Post-Suppression = S3, Post-Stimulation = S4) across the events (Infancy = Inf, Year One = Y1, Year Two = Y2), which are outlined in yellow boxes. The upper diagonal has Spearman’s correlation coefficients, while the directional skew and tightness of the ellipses in the lower diagonal provides a visual representation of the association.

**Supplementary Table 1.** Pairwise comparisons of variance for the raw plasma cortisol values, across sampling events and types. Samples are color-coded by year (Infant samples = green, Year 1 = yellow, Year 2 = red). Significance indicates between group differences in variation for raw cortisol values. Tests that involve across year comparisons of the same samples, as emphasized in the main text, are bolded. Note that *p* values are adjusted for multiple comparisons using a Holm-Bonferroni correction.

| **Level (a)** | **Level (b)** | ***p*-value** | **No difference** |
| --- | --- | --- | --- |
| Inf_Samp1 | Inf_Samp2 | 1.000 | Not Reject |
| Inf_Samp1 | Inf_Samp3 | 0.080 | Not Reject |
| **Inf_Samp1** | **Y1_Samp1** | **1.000** | **Not Reject** |
| Inf_Samp1 | Y1_Samp2 | 1.000 | Not Reject |
| Inf_Samp1 | Y1_Samp3 | <0.001 | Reject |
| **Inf_Samp1** | **Y2_Samp1** | **0.032** | **Reject** |
| Inf_Samp1 | Y2_Samp2 | <0.001 | Reject |
| Inf_Samp1 | Y2_Samp3 | <0.001 | Reject |
| Inf_Samp2 | Inf_Samp3 | 0.043 | Reject |
| Inf_Samp2 | Y1_Samp1 | 1.000 | Not Reject |
| **Inf_Samp2** | **Y1_Samp2** | **0.635** | **Not Reject** |
| Inf_Samp2 | Y1_Samp3 | <0.001 | Reject |
| Inf_Samp2 | Y2_Samp1 | 0.024 | Reject |
| **Inf_Samp2** | **Y2_Samp2** | **<0.001** | **Reject** |
| Inf_Samp2 | Y2_Samp3 | <0.001 | Reject |
| Inf_Samp3 | Y1_Samp1 | 0.016 | Reject |
| Inf_Samp3 | Y1_Samp2 | 0.540 | Not Reject |
| **Inf_Samp3** | **Y1_Samp3** | **0.988** | **Not Reject** |
| Inf_Samp3 | Y2_Samp1 | 1.000 | Not Reject |
| Inf_Samp3 | Y2_Samp2 | 1.000 | Not Reject |
| **Inf_Samp3** | **Y2_Samp3** | **0.009** | **Reject** |
| Y1_Samp1 | Y1_Samp2 | 0.352 | Not Reject |
| Y1_Samp1 | Y1_Samp3 | <0.001 | Reject |
| **Y1_Samp1** | **Y2_Samp1** | **<0.001** | **Reject** |
| Y1_Samp1 | Y2_Samp2 | <0.001 | Reject |
| Y1_Samp1 | Y2_Samp3 | <0.001 | Reject |
| Y1_Samp2 | Y1_Samp3 | <0.001 | Reject |
| Y1_Samp2 | Y2_Samp1 | 0.244 | Not Reject |
| **Y1_Samp2** | **Y2_Samp2** | **<0.001** | **Reject** |
| Y1_Samp2 | Y2_Samp3 | <0.001 | Reject |
| Y1_Samp3 | Y2_Samp1 | 0.001 | Reject |
| Y1_Samp3 | Y2_Samp2 | 1.000 | Not Reject |
| **Y1_Samp3** | **Y2_Samp3** | **<0.001** | **Reject** |
| Y2_Samp1 | Y2_Samp2 | <0.001 | Reject |
| Y2_Samp1 | Y2_Samp3 | <0.001 | Reject |
| Y2_Samp2 | Y2_Samp3 | <0.001 | Reject |


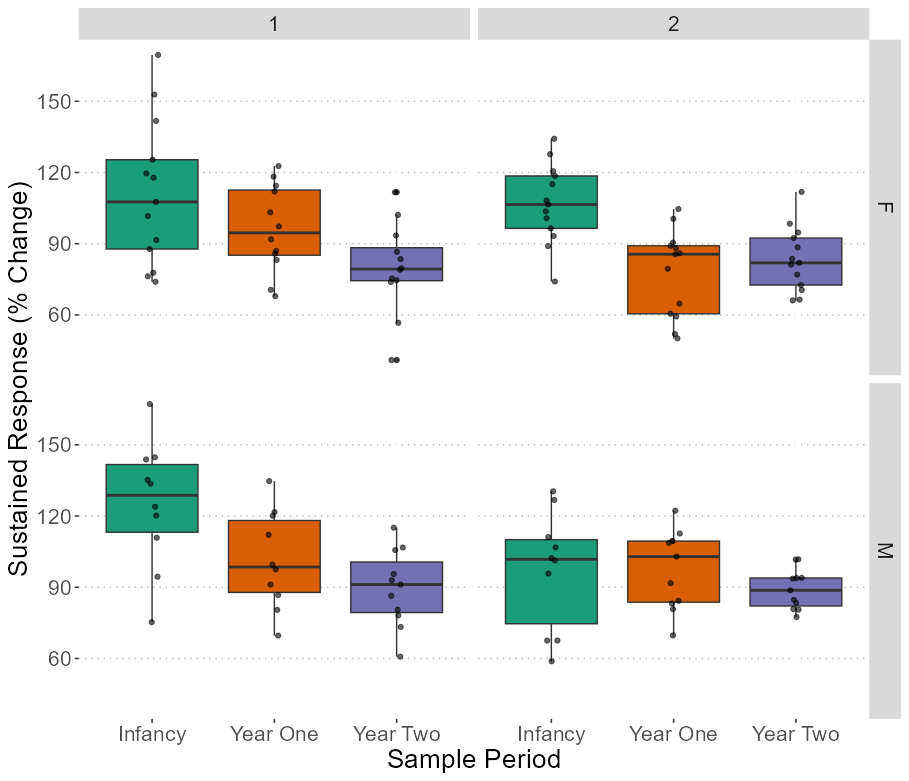


**Supplementary Figure 5**. Sustained response – percent change to sample point 2 from sample point 1. A boxplot for data presented in manuscript **Figure 3**, with panels dividing the data by sex (rows) and cohort (columns). Boxplots are overlaid with jittered datapoints; color emphasizes year of sampling.


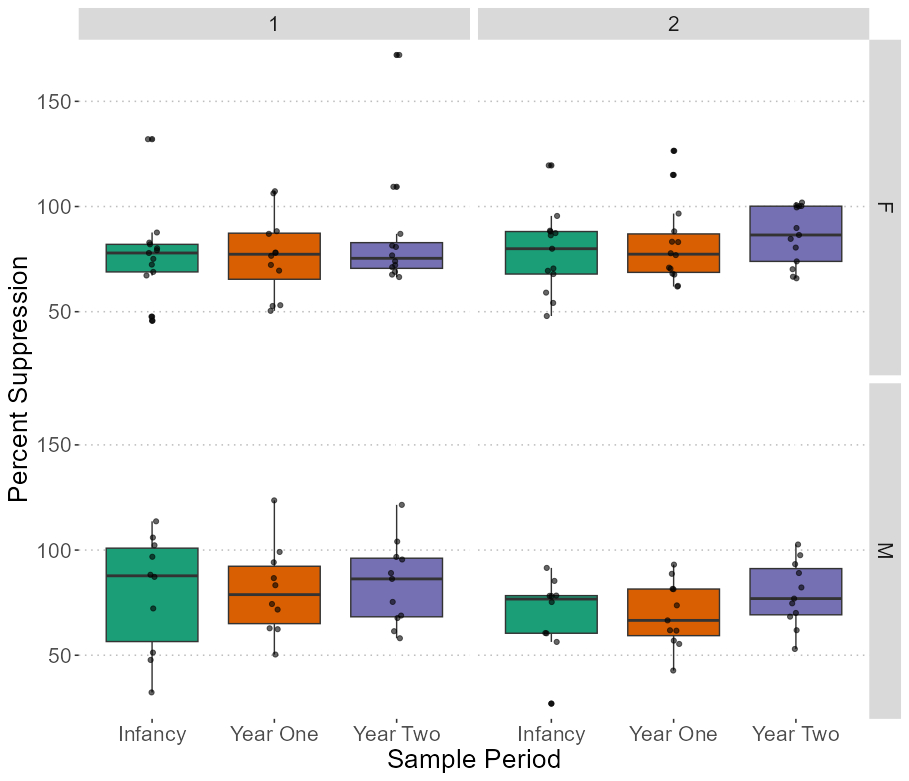


**Supplementary Figure 6**. Percent suppression of endogenous cortisol output by dexamethasone – percent change to sample point 3 from sample point 2. A boxplot for data presented in manuscript **Figure 4**, with panels dividing the data by sex (rows) and cohort (columns). Boxplots are overlaid with jittered datapoints; color emphasizes year of sampling.
